# Benchmarking of cell type deconvolution techniques for microarray data

**DOI:** 10.1016/j.isci.2026.118079

**Published:** 2026-10-29

**Authors:** Zoha Kamali, Harold Snieder, Ahmad Vaez

**Affiliations:** 1 Medical Image and Signal Processing Research Centre, Isfahan University of Medical Sciences, Isfahan, Iran; 2 Department of Bioinformatics, Isfahan University of Medical Sciences, Isfahan, Iran; 3 Department of Epidemiology, University Medical Centre Groningen, University of Groningen, Groningen, the Netherlands

**Keywords:** Cell-type deconvolution, microarray, single-cell RNA sequencing, benchmarking

## Abstract

Cell type deconvolution algorithms have significantly advanced in recent years. However, a notable gap remains regarding the performance of these algorithms when applied to microarray data, which is widely available in numerous disease contexts. Here we compare the performance of existing cell type deconvolution algorithms across a spectrum of data preparation scenarios and diverse cell types within the context of microarray-based bulk transcriptome. Among the ten algorithms evaluated, MuSiC, qprogwc, and CIBERSORTx emerged as the top performers, demonstrating the highest correlation between the estimated and real cell type compositions (*r*_max_ = 0.74, 0.69, and 0.67, respectively). Our study provides a practical framework for microarray deconvolution, while also underscoring the necessity for greater precision in these techniques. Such methods would unlock the full potential of the vast repositories of microarray data in public domains, facilitating deeper insights into cellular compositions in various disease states, and promoting translational research.

## Highlights

• Ten deconvolution algorithms were benchmarked across multiple analysis conditions
• MuSiC, qprogwc, and CIBERSORTx show superior deconvolution accuracy
• Rare cell types are harder to recover, but top methods remain the most accurate
• qprogwc combines high accuracy with computational efficiency

## Graphical abstract

**Figure:**
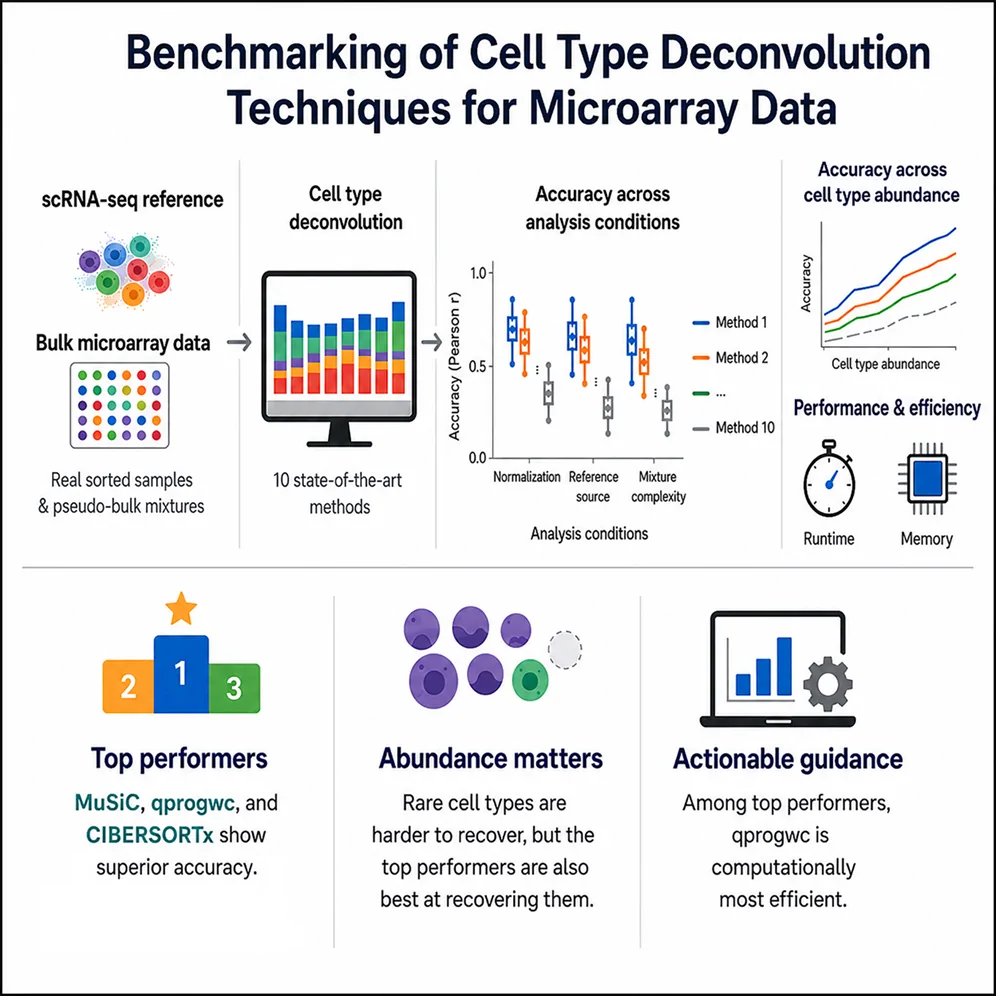

## Teaser

Biological sciences; Bioinformatics; Transcriptomics

## Introduction

Cells, as the fundamental units of life, are organized in intricate patterns to form tissues and organs, each with unique gene expression profiles and morphologies. Traditional bulk gene expression analysis methods, while useful for obtaining average population data, often obscure the nuanced properties of individual cells. This limitation has led to the development of single-cell RNA sequencing (scRNA-seq) technologies, which have significantly advanced our understanding of cellular heterogeneity. However, the higher costs of this technology limit its application in different disease conditions and for large sample sizes. On the other hand, there are vast amounts of microarray-based bulk gene expression data available in different disease conditions and with large sample sizes that can still be used in basic and translational research.

Cell type deconvolution techniques aim to estimate the cell-type composition within these mixed populations in bulk data, thereby enabling researchers to dissect the contributions of individual cell types to the overall gene expression profile of the tissue.^1^^,^^2^^,^^3^ This is particularly important in the study of diseases where the cellular composition can significantly influence disease progression and response to treatment. Hence, cell type deconvolution is crucial to advance our understanding of biological processes and disease mechanisms.^4^^,^^5^^,^^6^^,^^7^

Current methods for cell type deconvolution leverage computational algorithms to infer cell-type proportions from bulk data.^1^^,^^8^^,^^9^^,^^10^ Benchmarking studies have been conducted to evaluate their performance under different conditions, such as data preparation methods, varying levels of noise, cell type heterogeneity, and the presence of rare cell types.^11^^,^^12^^,^^13^ The integration of machine learning techniques, such as ensemble learning and deep learning, has also been explored to improve the accuracy and robustness of deconvolution methods.^8^^,^^10^^,^^14^ These approaches can potentially capture complex patterns in the data that traditional algorithms might miss.

However, an important knowledge gap is yet to be bridged. Most recent methods and benchmarking studies focus on bulk RNA-seq datasets, with the opportunity to use single-cell RNA-seq data as the reference for cell type estimation. However, most deconvolution methods developed for microarray data rely on a limited set of pre-defined signature matrices. A signature matrix is a reference file that assigns specific genes and their weights to each cell type, allowing the discrimination of cell subsets within a tissue and the estimation of cell-type proportions in bulk transcriptomes. Unfortunately, signature matrices for microarray deconvolution are usually generated from Fluorescence-Activated Cell Sorting (FACS)-separated datasets, which are generally available for blood, limiting their broader applicability. Although single-cell transcriptomic resources allow the construction of broader signatures, platform differences mean that such signatures require additional calibration and validation before being integrated into microarray deconvolution. On the other hand, microarray datasets are still more cost-effective to generate and—even more importantly—are among the most widely available. An exemplar is the Gene Expression Omnibus (GEO) database.^15^ This database currently includes omics data from about seven million samples of different species, of which about four million are human. In human gene expression profiles, 41% of the submitted datasets are still of the array type, representing samples of many different disease conditions. This is a strong argument for leveraging this kind of data to address questions on cell type composition in health and disease and microenvironments, and to promote our understanding of the underlying biological processes and disease mechanisms.

Here we provide benchmarking of 10 state-of-the-art cell type deconvolution techniques that we compared using the same input microarray-based bulk dataset as well as the same scRNA-seq reference panel for model training. Importantly, the bulk dataset is derived from FACS-separated samples, so the true cell type composition of each sample is known. This allows us to quantitatively compare the performance of different methods. We performed comparisons across various data preparation procedures for both bulk and reference datasets, tested different strategies for selecting probes in multi-probe genes to match the reference, and evaluated combinations of optimal conditions for microarray-based cell type deconvolution. The overall study design is illustrated in Figure 1, with a simplified schematic overview provided in Figure S1. The main components of the study are described in Figure S2.

**Figure 1:**
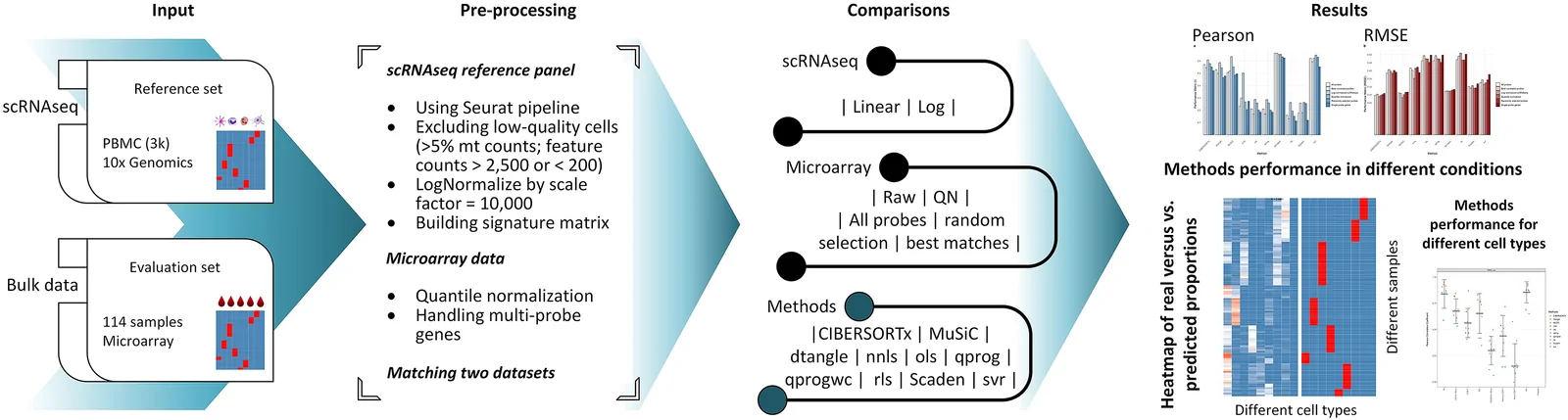
Schematic of overall study design PBMC: 2,700 peripheral blood mononuclear cells from the 10× Genomics website, used as the single-cell RNA sequencing reference panel. Microarray dataset was used as bulk transcriptome input. This dataset includes 114 samples with known cell type compositions using FACS technology. QN: Quantile normalized, RMSE: root mean squared error.

## Results

Comparing the linear and log-transformed scRNAseq reference datasets confirmed that both versions are of good quality and valid for use based on the following criteria a) cell type similarity matrix: in both dataset versions expression measures of similar blood cell types were almost correlated to each other (e.g., memory and naive CD4 T cells showed *r* > 0.6, and FCGR3A+ and CD14^+^ monocytes showed *r* > 0.5 in both dataset versions), and b) hierarchical clustering: relevant cell types were clustered together after applying hierarchical clustering algorithm, with the log-transformed version making even better sense by showing both monocyte groups of CD14^+^ and FCGR3A + into one cluster (Figure 2A –bottom section).

**Figure 2:**
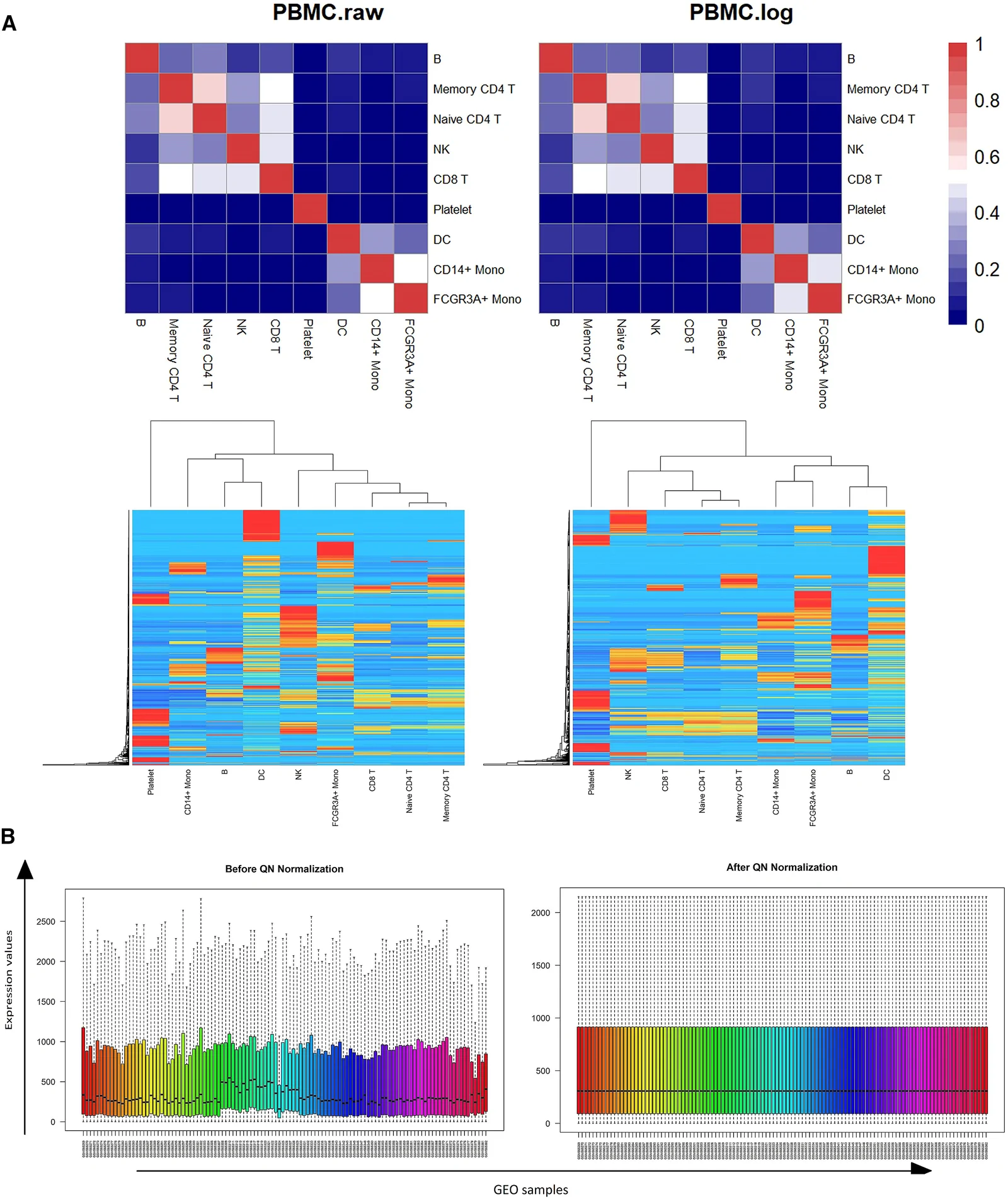
Data preparation visualizations that show the validity of bulk microarray as well as scRNA-seq reference datasets (A) Pairwise correlation and hierarchical clustering of cell type expression profiles in peripheral blood mononuclear cells (PBMC) reference in both raw linear and log-transformed scales. (B) bulk microarray data before (left) and after (right) quantile normalization (QN). B: B lymphocytes, NK: Natural Killer cells, DC: Dendritic Cells, Mono: Monocyte cells.

Quantile normalization of the microarray data generated a uniform and comparable distribution of gene expression data across samples (Figure 2B). In the matching procedure of the cell types of microarray samples with those of the scRNA-seq reference, an inconsistency was perceived: only one group of monocytes was available in microarray samples, while two separate monocyte cell types were present in the scRNA-seq reference, i.e., CD14^+^ and FCGR3A + monocytes. To match these cell types in bulk and reference datasets, we separated microarray samples of monocyte cell types into two groups based on the distribution of *CD14* and *FCGR3A* gene expression levels. An empirical threshold of *CD14* raw expression value over 13000 was considered to assign monocytes into the CD14^+^ subtype (Figure S3).

Among data preparation conditions, using all probes and QN microarray data was better overall (Tables 1 and 2), while reducing this data to only single-probe genes reduced the performance across all methods (Figure 3A). For the scRNAseq reference, using the log-transformed data resulted in the best performance overall (Table 3).

**Table 1:** Number of cell type deconvolution algorithms in which each probe selection strategy gets the maximum performance among other probe selection strategies

Criterion	All probes	Best probe selection	Random probe selection	Only single-probe genes
Correlation r	6	3	1	0
RMSE^a^	4	2	3	1

RMSE: Root-Mean-Square Error.

a The maximum performance means the highest r but the lowest RMSE.

**Table 2:** Number of cell type deconvolution algorithms in which each microarray data preparation strategy gets the maximum performance among other microarray data preparation strategies

Criterion	microarray data before quantile normalization	Quantile-normalized microarray data
Correlation r	1	9
RMSE^a^	3	7

RMSE: root-mean-square error.

a The maximum performance means the highest r but the lowest RMSE.

**Figure 3:**
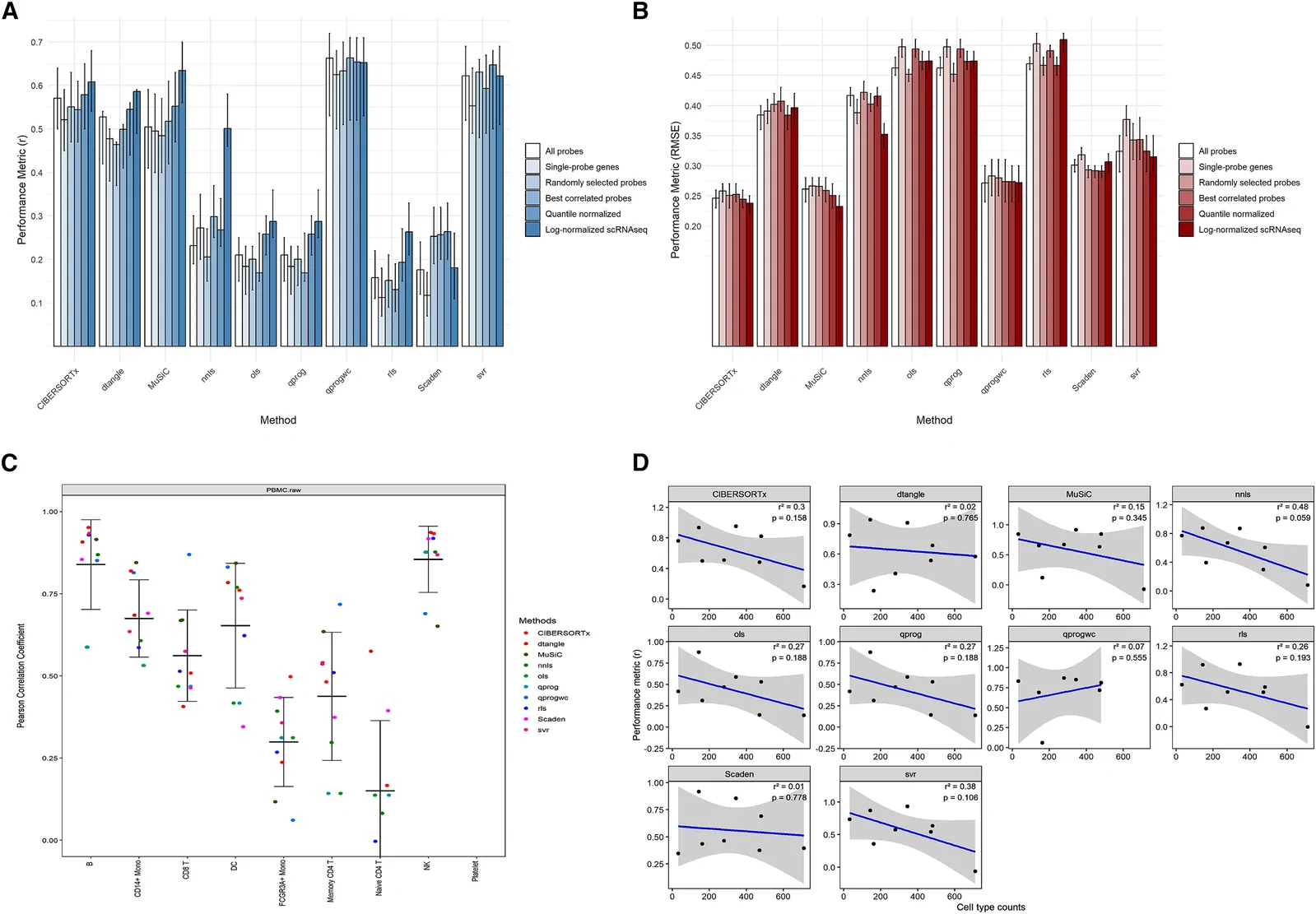
Comparison of prediction performance of different algorithms under different data preparation conditions and across different cell types (A) correlation coefficients (Pearson’s r) and B) root mean squared error (RMSE) of real versus estimated cell type proportions for different algorithms under different data preparation conditions i.e., 1–5) linear scRNAseq data as the reference and five versions of microarray data (i.e., raw data using all probes, using only single-probe genes, using also multi-probe genes but upon random probe selection to match the reference gene, using also multi-probe genes but based on best gene expression correlation with the scRNAseq reference, and quantile-normalized (QN) data of all probes) as the input bulk datasets, and 6) log-normalized scRNAseq data as the reference and raw microarray data of all probes as the input bulk dataset. The error bars represent the 95% confidence interval (CI95%) calculated using the bootstrap method. (C) Prediction performance of different algorithms across different cell types. Negative correlation values were not shown. (D) the correlation of prediction performance with cell type coverage in the scRNA-seq reference panel.

**Table 3:** Number of cell type deconvolution algorithms in which each single-cell RNA-seq reference data preparation strategy gets the maximum performance among other reference data preparation strategies

Criterion	scRNA-seq data before log-normalization	Log-normalized scRNAseq data
Correlation r	1	9
RMSE^a^	5	5

RMSE: root-mean-square error.

a The maximum performance means the highest r but the lowest RMSE.

Comparisons of the performance of different cell type deconvolution algorithms in estimating cell type proportions in bulk microarray samples showed the highest Pearson’s correlations (*r* > 0.5) across different conditions for CIBERSORTx, dtangle, MuSiC, qprogwc, and svr (Figure 3A). Of these, CIBERSORTx, MuSiC, and qprogwc also showed the lowest root mean squared error (RMSE), i.e., <0.3 (Figure 3B; Figure S4). Statistical analyses between methods were also supportive of this conclusion (Tables S1, S2, and S3). Simulated pseudo-bulk samples also showed the best performance for CIBERSORTx, MuSiC, and qprogwc (Figure S5).

For different cell types, the performance of cell type deconvolution showed large variation, with the highest median correlation (r) for qprogwc and MuSiC, and the highest mean correlation for qprogwc and CIBERSORTx (Figure 3C; Table 4). The coverage of cell types in the reference showed some impact on the performance of predictions; however, this was not statistically confirmed (all correlation *p* values being non-significant, i.e., >0.05) (Figure 3D). On the other hand, stratifying mixed samples into cell type abundance groups showed that most algorithms perform less accurately when estimating rare cell populations compared with more prevalent ones (Figure S6). Among the evaluated methods, CIBERSORTx, MuSiC, and qprogwc exhibited the lowest estimation errors overall.

**Table 4:** Comparison of different cell type deconvolution algorithms based on correlation coefficients of their estimated vs. real cell type proportions, separately in different cell types

Method	Mean performance	Median performance	# Maximum performance^a^	Maximum performance
qprogwc	0.69	0.81	2	0.87
MuSiC	0.58	0.66	2	0.92
CIBERSORTx	0.64	0.63	2	0.95
dtangle	0.63	0.63	2	0.94
nnls	0.57	0.64	0	0.88
svr	0.57	0.61	0	0.93
rls	0.54	0.55	0	0.93
Scaden	0.56	0.45	0	0.92
ols	0.43	0.44	0	0.88
qprog	0.43	0.44	0	0.88

The table is sorted based on 1) # maximum performance and 2) median performance.

a Number of cell types in which the method gets maximum performance.

Final comparisons of the three prioritized methods, i.e., MuSiC, qprogwc, and CIBERSORTx, under the optimal combination of data preprocessing settings for both microarray and scRNA-seq reference data, showed that MuSiC achieved the highest overall correlation with the true cell type proportions (Pearson’s *r* = 0.74) (Figure 4).

**Figure 4:**
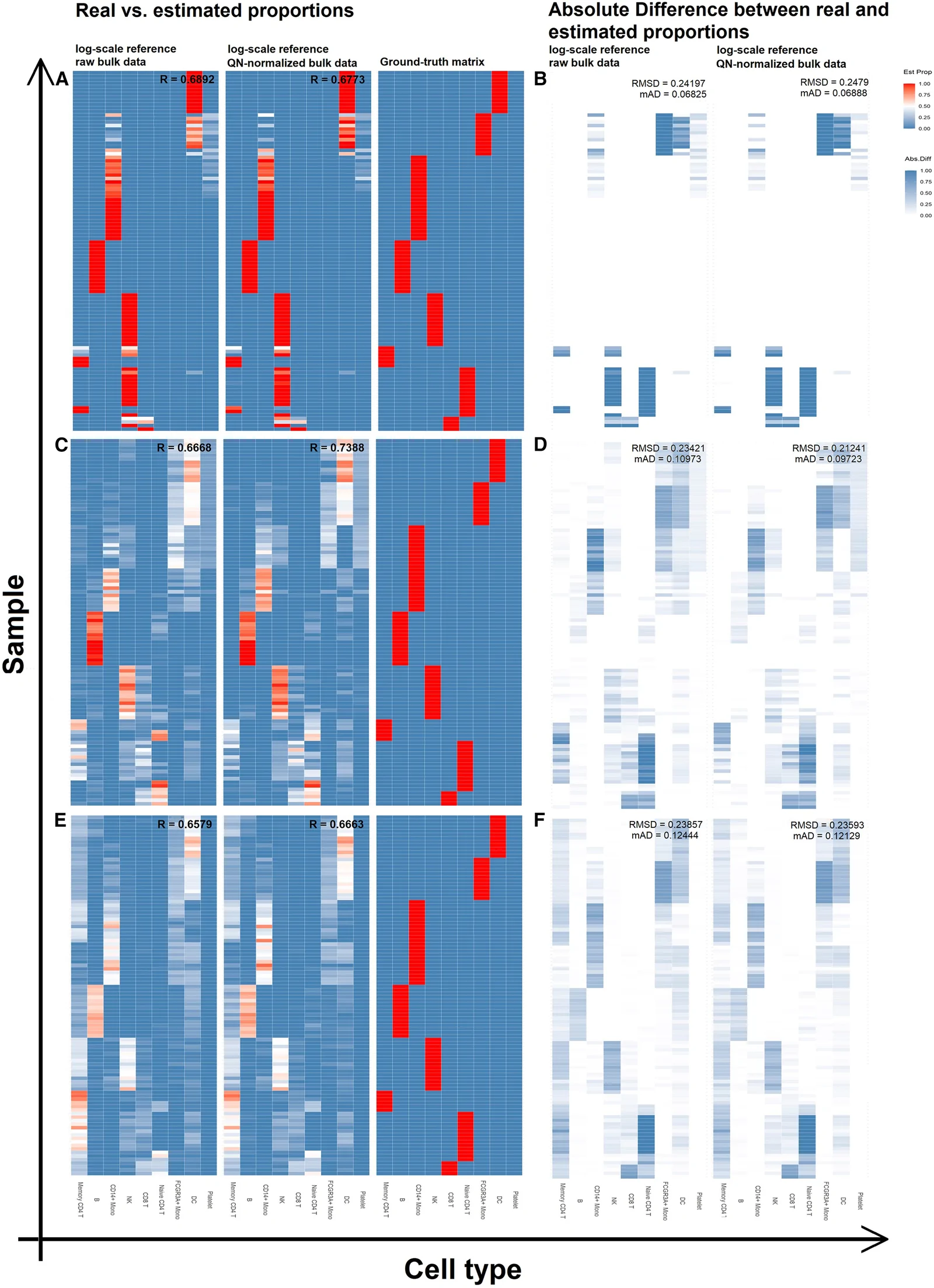
Comparison of the prediction performance for the top three performers qprogwc (A and B), MuSiC (C and D), and CIBERSORTx (E and F) were compared when using a raw microarray dataset or a QN-normalized microarray dataset with the log-transformed scRNA-seq reference panel. The left three-part panels are the estimated (two left parts) versus real (the right-hand part) cell type proportions, and the right two-part panels are the absolute difference between these two values for each sample.

Performance comparisons when changing the single-cell reference also supported the three above top performers, while showing an acceptable performance for dtangle, Scaden, and svr (Figure S7). However, when considering runtime and memory usage, methods such as CIBERSORTx, svr, MuSiC, and Scaden position as less computationally efficient options compared to others that demand less time and memory (Figure S8).

## Discussion

We performed a comparative study of the prediction performance of different state-of-the-art cell type deconvolution algorithms for bulk datasets generated by microarray technology. We based comparisons on different data preparation techniques for the bulk microarray dataset as well as the single-cell RNA sequencing reference, and different cell types.

Among the evaluated methods, MuSiC, qprogwc, and CIBERSORTx demonstrated superior performance, yielding the highest correlation coefficients (*r* > 0.5) and the lowest root mean squared errors (RMSE < 0.3). The best overall prediction accuracy was achieved by the MuSiC pipeline (*r* = 0.74; RMSE = 0.21) when applied to QN microarray data using a log-transformed scRNAseq reference panel.

We also showed that prediction performance varies substantially across cell types (Figure 3C), with correlation coefficients ranging from −0.07 (Naive CD4 T cells) to 0.95 (B cells), and this variability was not statistically confirmed to be a function of the corresponding cell-type abundance in the reference (all correlation *p* values >0.05) (Figure 3D). Overall, the three best-performing methods (MuSiC, qprogwc, and CIBERSORTx) yielded high correlations (median r ≈ 0.7) across different cell types.

Strengths of our study include i) focusing on microarray bulk data in cell type deconvolution for the first time, ii) assessing the effects of multi-probe genes on prediction performance by designing different probe-gene matching scenarios, iii) evaluating the performance across different cell types, and iv) showing the performance for a wide range of deconvolution techniques from more classical to machine learning- and artificial intelligence-based algorithms.

In our simulations, we generated mixed microarray data. It is important to acknowledge that these simulations provide an approximation of real-world scenarios. Microarray data are based on intensity readings rather than read counts, which means the underlying distribution is not strictly linear. Consequently, deviations from linearity may occur at very low or very high intensity levels.

Although our analysis focuses on comparing deconvolution performance under commonly used preprocessing steps for input datasets, we acknowledge that alternative normalization, transformation, or marker gene selection strategies may influence prediction performance. A systematic evaluation of the best preprocessing choices for the top performers recommended here would require a dedicated follow-up study, which lies beyond the scope of the present work.

Another consideration is large variation in prediction performance across almost all methods for different cell types, which could not be attributed to variation in coverage depth in the scRNAseq reference, suggesting other biological or technical factors are at play. A particular example is the lower performance observed for Naive CD4 T cells compared to other cell types. Potential reasons for this reduced performance may include limitations in the quality or representativeness of the reference profiles used for Naive CD4 T cells, which may not fully capture their unique gene expression patterns, or intrinsic characteristics of Naive CD4 T cells themselves, such as less distinct marker gene expression, that pose challenges for current deconvolution algorithms. This observation also highlights the broader challenge that deconvolution tools face when attempting to resolve closely related or lineage-proximal cell types. In addition to marker-gene specificity and the quality of reference data, the ability to accurately distinguish such populations also relies on the underlying assumptions of the algorithmic models. Further research is clearly needed to explore the effects of cell-type heterogeneity and similarity on prediction performance. Investigating performance across controlled gradients of cell-type similarity would require specially designed datasets and constitutes an important, yet separate, methodological question that is beyond the scope of this current study.

More research is needed to explore the effects of cell type variety in prediction performance. This may also interact with the ability of deconvolution tools to resolve closely related or lineage-proximal cell types. Distinguishing such populations typically depends on marker-gene specificity, reference quality, and algorithmic modeling assumptions. Investigating performance across controlled gradients of cell-type similarity would require specially designed datasets and constitutes an important but separate methodological question.

Similarly, the intrinsic differences between microarray and RNA-seq platforms—including dynamic range, background noise, probe hybridization behavior, and detection sensitivity—can contribute to performance variability across tools. Dissecting the individual effects of these platform-specific factors would require targeted experimental or computational designs. We highlight these aspects as important directions for future methodological work but outside the primary aim of this study, which was to characterize the performance of established deconvolution tools on real microarray data with commonly defined cell-type categories.

There is still a strong need to develop specific methods for microarray data. A significant performance gap remains between current methods for microarray data and those reported in previous RNA-seq studies—i.e., correlations of estimated and real proportions based on our findings are often below 0.75 for microarrays, compared to over 0.9 for RNA-seq in previous studies.^11^ Nonetheless, this difference should be interpreted with caution, as it is an indirect comparison rather than a head-to-head evaluation of microarray and RNA-seq using the same samples and the same single-cell reference. Several technical factors may contribute to this discrepancy. Microarray platforms have a more limited dynamic range, are prone to signal saturation and cross-hybridization, and may miss low-expressed or cell-type-specific marker genes that are captured by RNA-seq. Moreover, the gene coverage of microarray platforms often does not fully overlap with marker sets derived from scRNA-seq, potentially reducing the effective resolution of deconvolution. Differences in noise structure, normalization procedures, and batch effects between microarray and RNA-seq can further affect model performance. Together, these factors may explain why algorithms that perform well in RNA-seq-based benchmarks show reduced accuracy when applied to microarray data using scRNA-seq-derived references.

In conclusion, cell type deconvolution holds great potential to provide biological insights into cellular functions and interactions in both healthy and diseased states.^11^^,^^14^^,^^16^ Although single-cell RNA sequencing now provides direct cell-level resolution, cell type deconvolution remains essential for the analysis of large-scale bulk transcriptomic datasets that dominate public repositories, as well as for contexts where single-cell profiling is impractical due to cost, tissue availability, or preservation constraints. Moreover, deconvolution complements single-cell data by enabling cross-study comparisons and validation in independent cohorts. Within this context, cell type deconvolution is becoming an indispensable analytical approach for systems-level interpretation of transcriptomic data.

The ongoing development of more sophisticated and accurate deconvolution methods will be pivotal in exploiting the full potential of transcriptomic data.^17^^,^^18^ Researchers are increasingly able to integrate bulk and single-cell data to unravel the complexities of tissue architecture, leading to more precise models of cellular behavior and interactions.^19^^,^^20^^,^^21^^,^^22^^,^^23^ This, in turn, is paving the way for diagnostic tools, targeted therapies, and personalized medicine approaches that consider the unique cellular composition of individual patients’ tissues.^24^^,^^25^

### Limitations of the study

Our study may be complemented by future efforts in several respects: i) we were able to test only one microarray dataset for our evaluations because of the limited availability of FACS-separated bulk microarray data with known cell types for each sample. Future work may consider other datasets and tissues; ii) other hypotheses for the variability of prediction performance in different cell types should be investigated to explore the causes and improve the results of prediction algorithms; iii) in order to fully preserve the comparability of the applied algorithms and the potential of using every tissue-specific scRNA-seq dataset as the reference, we included reference-based algorithms which could accept the reference as input. We excluded methods based on other data types (e.g., spatial transcriptomics)^8^^,^^10^ or those geared toward a specific tissue or disease.^26^ Future efforts may also focus on reference-free algorithms, or those based on predefined references such as xCell,^24^ while assuring the comparability of results.

## Resource availability

### Lead contact

Requests for further information and resources should be directed to and will be fulfilled by the lead contact, Dr. Ahmad Vaez (a.vaez@umcg.nl).

### Materials availability

This study did not generate new unique reagents.

### Data and code availability

• The microarray dataset comprising 114 samples of blood cell types is publicly available through the Gene Expression Omnibus (GEO) under accession number GEO: GSE22886. The single-cell RNA-seq reference datasets are publicly available from 10x Genomics (PBMC3k and PBMC8k datasets at https://www.10xgenomics.com/).
• All tools used in this study are publicly available as R packages, standalone software, or web-based tools. CIBERSORTx is available at https://cibersortx.stanford.edu/. MuSiC and Scaden are available on GitHub at https://github.com/xuranw/MuSiC and https://github.com/KevinMenden/scaden, respectively. The other deconvolution algorithms are available through the granulator R package at https://github.com/Novartis/granulator.
• Any additional information required to reanalyze the data reported in this paper is available from the lead contact upon request.

## Acknowledgments

Funding support is provided by 10.13039/501100003970Isfahan University of Medical Sciences, Isfahan, IR (grant no 2403356) to Dr. Ahmad Vaez and Dr. Zoha Kamali.

We thank the Center for Information Technology of the University of Groningen for their support and for providing access to the Hábrók high-performance computing cluster.

## Author contributions

Z.K.: conceptualization, methodology, validation, formal analysis, investigation, resources, data curation, writing – original draft, and visualization. A.V.: supervision, project administration, writing – review and editing, and funding acquisition. H.S.: supervision, project administration, and writing – review and editing.

## Declaration of interests

The authors declare that they have no competing interests.

## Declaration of generative AI and AI-assisted technologies in the manuscript preparation process

During the preparation of this work, the authors used ChatGPT (OpenAI) to improve language and readability, refine the writing, and generate a graphical abstract. After using this tool, the authors reviewed and edited the content as needed and took full responsibility for the content of the publication. All scientific content, data analysis, plots, figure interpretations, and conclusions in this manuscript were generated entirely by the authors without the use of generative AI tools.

## STAR★Methods

### Key resources table

**Table:** 

REAGENT or RESOURCE	SOURCE	IDENTIFIER
**Deposited data**		

The main scRNAseq reference panel for benchmarking	10x Genomics data repository https://cf.10xgenomics.com/samples/cell/pbmc3k/pbmc3k_filtered_gene_bc_matrices.tar.gz	PBMC3k
The validation scRNAseq reference panel	10x Genomics data repository https://cf.10xgenomics.com/samples/cell-exp/2.1.0/pbmc8k/pbmc8k_filtered_gene_bc_matrices.tar.gz	PBMC8k
The bulk microarray dataset of 114 samples	GEO database https://www.ncbi.nlm.nih.gov/geo/query/acc.cgi?acc=GSE22886	GEO: GSE22886

**Software and algorithms**		

CIBERSORTx	https://cibersortx.stanford.edu/	N/A
MuSiC	https://github.com/xuranw/MuSiC	N/A
Scaden	https://github.com/KevinMenden/scaden	N/A
granulator	https://github.com/Novartis/granulator	N/A

### Experimental model and study participant details

This study is a computational analysis of previously generated and publicly available transcriptomic data and did not involve recruitment or direct interaction with human participants. This work has been approved by Research Ethics Committees of Isfahan University of Medical Sciences and Health Services (IR.MUI.REC.1404.017).

### Method details

#### Input data

##### scRNAseq reference panel

Standard peripheral blood mononuclear cells (PBMC) of 2,700 cells from a healthy human donor, generated by 10x Genomics technology (available at cf.10xgenomics.com)^27^ and used as tutorial in the Seurat pipeline^19^ (https://satijalab.org/seurat/articles/pbmc3k_tutorial), was used as the scRNAseq reference to estimate cell type compositions in bulk data (download link: https://cf.10xgenomics.com/samples/cell/pbmc3k/pbmc3k_filtered_gene_bc_matrices.tar.gz). For independent validation, PBMC data of 8k cells from another healthy donor was used from the same resource (download link: https://cf.10xgenomics.com/samples/cell-exp/2.1.0/pbmc8k/pbmc8k_filtered_gene_bc_matrices.tar.gz).

##### Microarray data

For the purpose of benchmarking different cell type deconvolution algorithms, when applied on bulk microarray data, we needed microarray samples with known cell type compositions to compare the true versus estimated cell type compositions. Hence we used a microarray dataset comprising 114 peripheral blood and bone marrow samples from normal human donors, representing nine broad cell types. Each cell type was isolated and purified by FACS technology^28^ (GEO: GSE22886). These nine cell types—B cells, CD4^+^ T cells, CD8^+^ T cells, NK cells, monocytes, neutrophils, dendritic cells, plasma cells, and memory T cells—were the only ones available in the dataset and therefore defined the scope of our benchmarking analysis.

#### Pre-processing

##### scRNAseq reference panels

After applying the standard data pre-processing steps of removing low-quality cells using the Seurat R package^19^ (nFeature_RNA >200 & nFeature_RNA <2500 & percent.mt < 5), two versions of data were prepared: i) raw counts, and; ii) log-normalized counts with a scale factor of 10,000 by default. These two versions were first assessed using the cell-type similarity matrix from the granulator R package^29^ and heatmap and hierarchical clustering provided by CIBERSORTx.^17^ These two reference datasets were then used to build prediction references for performance comparisons of the cell type deconvolution algorithms (Supplementary Material).

##### Microarray data

Two main parameters were considered to affect prediction accuracy: data normalization and how to treat genes tagged by multiple probes (we call these ‘multi-probe genes' from now on) when matching bulk data with the reference scRNAseq data. Hence, we prepared five versions of this data: i) raw data of all probes, ii) raw data of single-probe genes, iii) raw data of single-probe genes and randomly selected probes for multi-probe genes, iv) raw data of single-probe genes and best correlating probes between microarray and reference scRNAseq selected for multi-probe genes, and v) quantile normalized (QN) data of all probes. In all analyses, the input microarray expression values were in linear scale, as provided by GEO. Selection strategy of best correlating probes is described in Supplements. Quantile normalization was applied using normalizeBetweenArrays from the limma R package.^30^ We then used this bulk data with known cell types to compare the performance of different algorithms to predict cell type composition of these samples.

##### Matching two datasets

We then tried to reduce the effects of differences in input and reference datasets on the methods comparisons by matching two datasets in terms of cell types and genes. First, microarray samples of cell types that were not covered by the scRNAseq reference were removed and 102 samples remained. Second, the rest of the cell types were investigated to exactly match with the reference one-by-one. If bulk samples represented a broad cell type while the reference contained more specific subtypes, we separated the bulk samples into corresponding subtypes based on their expression profiles to match the resolution of the reference. Third, probes were matched to genes based on the accompanying platform annotation file, i.e., GPL96, provided by the GEO database. We used all common genes between the two datasets for deconvolution based on each method’s default parameters.

#### Comparison of the methods’ performance

##### Analysis conditions

The analyses conditions under which different methods were compared were designed to: 1) select best version of reference data from linear or log-transformed, and; 2) select the best version of microarray data with regard to the probe-matching and quantile normalization status. For the first purpose, two conditions were compared: a) linear scRNAseq data (i.e., filtered data before log-transformation) as the reference and raw microarray data as the input bulk dataset, and; b) log-normalized scRNAseq data as the reference and raw microarray data as the input bulk dataset. For the second purpose, five conditions were compared where linear scRNAseq data is used as the reference and five abovementioned versions of microarray data were used as the input bulk datasets.

After preparing and matching the bulk and scRNAseq data, the performance of ten cell type deconvolution algorithms including MUlti-Subject Single Cell deconvolution (MuSiC),^7^ the extended version of Cell type Identification By Estimating Relative Subsets Of Transcription (CIBERSORTx),^17^ single cell-assisted deconvolutional deep neural networks (Scaden),^14^ non-negative least-squares regression model (nnls),^31^ ordinary least squares (ols),^29^ Quadratic programming without constraints (qprog) and Quadratic programming with non-negativity and sum-to-one constraint (qprogwc),^32^ Linear mixing model (dtangle),^33^ Re-weighted least squares (rls),^34^ and Support vector regression (svr),^3^ were compared across six conditions: 1–4) raw counts of scRNAseq reference and raw bulk data of all probes, only single-probe genes, single-probe genes plus randomly selected probes for multi-probe genes, or single-probe genes plus best correlating probes between microarray and reference scRNAseq selected for multi-probe genes, 5) raw counts of scRNAseq reference and quantile normalized (QN) bulk data of all probes, and 6) log-normalized counts of scRNAseq reference and raw bulk data of all probes. We also compared the performance of these algorithms in the most basic form (raw bulk and raw reference) across different cell types and explored whether the coverage of cell types in the reference impacts the performance of predictions. Finally, we repeated comparisons for the best performing methods in the first round of comparisons, this time with a combination of data versions with best performance.

Pearson correlation coefficient (r) between the real and estimated cell type proportions as well as root-mean-square error (RMSE) of these estimations were the two criteria for these comparisons.

For independent validation, all these comparisons were repeated using the PBMC8k scRNAs-seq dataset.

##### Simulating pseudo-bulk mixtures

We also generated 1000 pseudo-bulk mixtures of the input microarray data to assess the deconvolution algorithms in samples more similar to the real conditions, where they are a mixture of cell types rather than purified cell types. Since microarray-based data is a projection of gene expression counts as expression intensity, we calculated the bulk expression using a weighted summation of expression in different cell proportions:

$${GE}_{x}^{\left(i\right)}=\sum_{k=1}^{n}P_{i.k}\times {GE}_{x.k}$$

Where ${GE}_{x}^{\left(i\right)}$ represents the simulated bulk gene expression value for gene x in sample i, n is the total number of cell types contributing to the mixture, *P*_*i*.*k*_ denotes the proportion of cell type k within sample i, and *GE*_*x*.*k*_ is the gene expression value of gene x specifically within cell type k.

We did not assume a fixed expression profile for each cell type across simulated mixtures (*GE*_*x*.*k*_). For each cell type in each mixture, one FACS-sorted microarray sample from that cell type was randomly selected from the available samples. Thus, different simulated mixtures could contain different experimentally observed expression profiles for the same cell type, thereby incorporating empirical sample-to-sample variation.

To introduce additional gene-level stochastic variation, the resulting mixture profile was subsequently perturbed using a multiplicative log-normal error term:

$${{\text{GE}}^{\prime }}_{x}^{\left(i\right)}={\text{GE}}_{x}^{\left(i\right)}\times \exp\left(\epsilon_{x}^{\left(i\right)}\right)$$

where $\epsilon_{x}^{\left(i\right)}∼\;N\left(0,\sigma^{2}\right)$ with (σ = 0.01). This approach introduces positive, gene-specific multiplicative variation while preserving the underlying expression signal and known cell-type proportions.

The reference signature matrix used by the deconvolution methods was the original PBMC3k scRNA-seq reference dataset.

#### Comparing run time and memory usage

In order to help decide which algorithm best fits the user’s needs, we also compared the required runtime and peak memory usage for each algorithm, independently using the same input data. All methods executable in a local environment (R-based and command-line tools) were benchmarked under identical computational conditions.

### Quantification and statistical analysis

#### Performance evaluation

Deconvolution performance was evaluated by comparing the estimated cell-type proportions with the corresponding ground-truth proportions. For the FACS-sorted microarray samples, performance was assessed using the Pearson correlation coefficient (r) between the predicted and observed cell-type proportions and the root mean squared error (RMSE). Pearson correlation was used to quantify the agreement in relative cell-type proportions, whereas RMSE quantified the magnitude of prediction errors. For the pseudo-bulk analyses, the same performance metrics were calculated using the known proportions used to generate each synthetic mixture as the ground truth.

#### Bootstrap uncertainty estimation

To quantify uncertainty in performance metrics, a non-parametric bootstrap approach was applied at the sample level. For each method–condition combination, 1000 bootstrap replicates were generated by resampling samples with replacement. For each replicate, r and RMSE were recomputed, and 95% confidence intervals (CI95%) were derived using the empirical 2.5th and 97.5th percentiles.

#### Statistical comparison of deconvolution algorithms

The Friedman test was used to assess overall differences in performance across methods while accounting for repeated measurements across preprocessing conditions.

Following a significant Friedman test (*p* < 0.05), pairwise comparisons between methods were conducted using the Conover post-hoc test with appropriate adjustment for multiple comparisons (*p* < 0.001 i.e., 0.05/45 with 45 as the number of pairwise tests).

#### Analysis of cell-type abundance

For the pseudo-bulk mixtures, deconvolution performance was additionally evaluated according to the abundance of the corresponding cell type. Cell-type proportions were categorized into five abundance groups: ultra-rare (<0.01), rare (0.01–0.05), low (0.05–0.10), medium (0.10–0.20), and high (>0.20). Pearson correlation and RMSE were calculated separately within each abundance group to assess the effect of cell-type abundance on prediction performance.

#### Computational performance

Runtime and memory measurements were summarized across repeated runs, and the corresponding mean and variability estimates are reported in the relevant figure and supplementary material.

Each benchmark was repeated 11 times, with the first run discarded to reduce potential effects of initialization and subsequent 10 runs used for summary statistics.

## Supplemental information

Document S1. Figures S1–S8 and Tables S1–S3, and Method S1

Data S1. Source data underlying Figure 3d
