# Supplementary material for: Benchmarking of cell type deconvolution techniques for microarray data
Source: iScience. 2026 Oct 29;29(11):118079. doi: 10.1016/j.isci.2026.118079 (PMC13742846; doi:10.1016/j.isci.2026.118079)
Supplement: Supplementary file 1 [file mmc1.pdf]

iScience, Volume *29*

Supplemental information

Benchmarking of cell type deconvolution techniques for microarray data

Zoha Kamali, Harold Snieder, and Ahmad Vaez

Supplementary figures

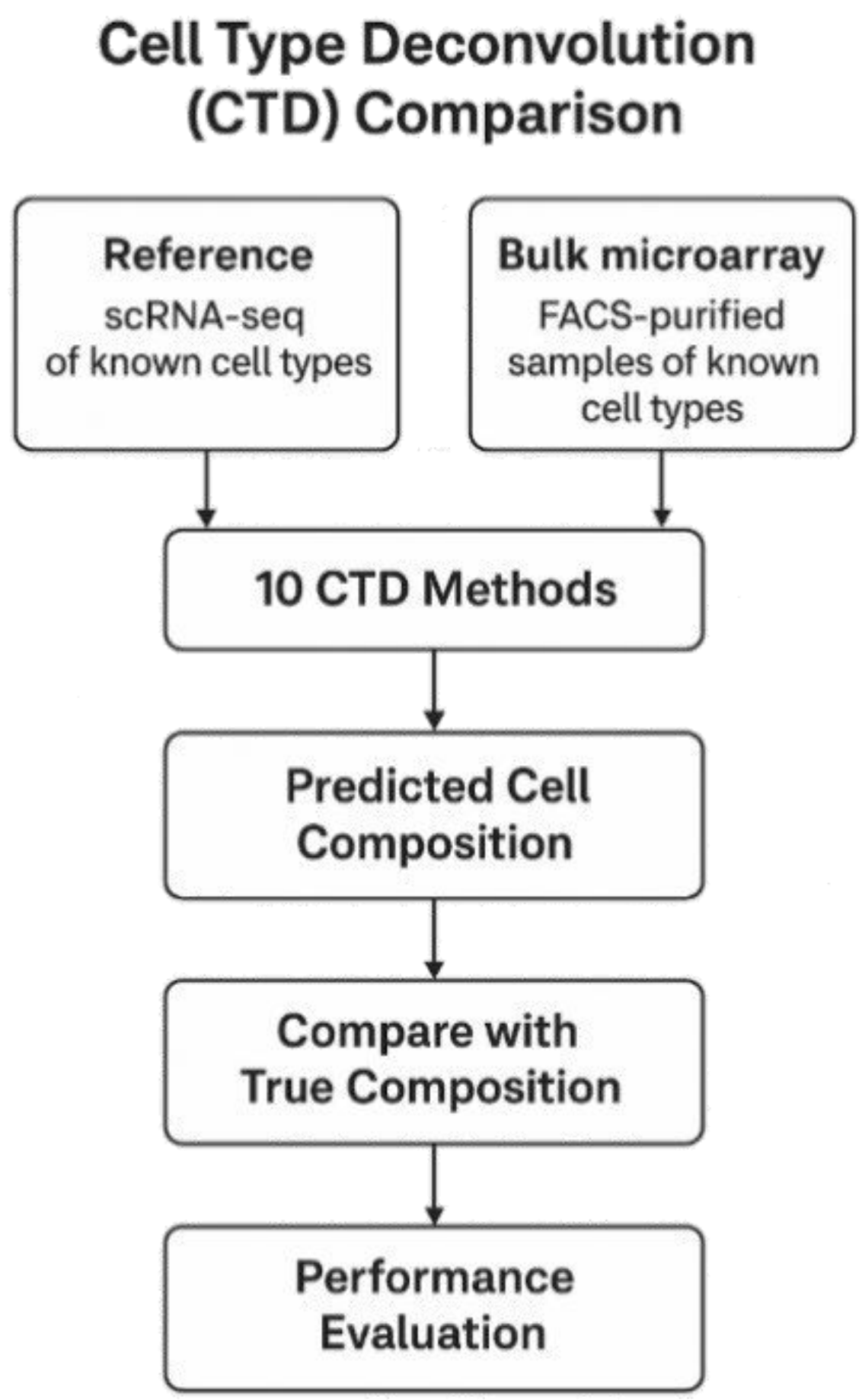

Figure S1. **A simplified schematic overview of the study design**. scRNA-seq: single-cell RNA sequencing dataset; FACS: Fluorescence-Activated Cell Sorting.

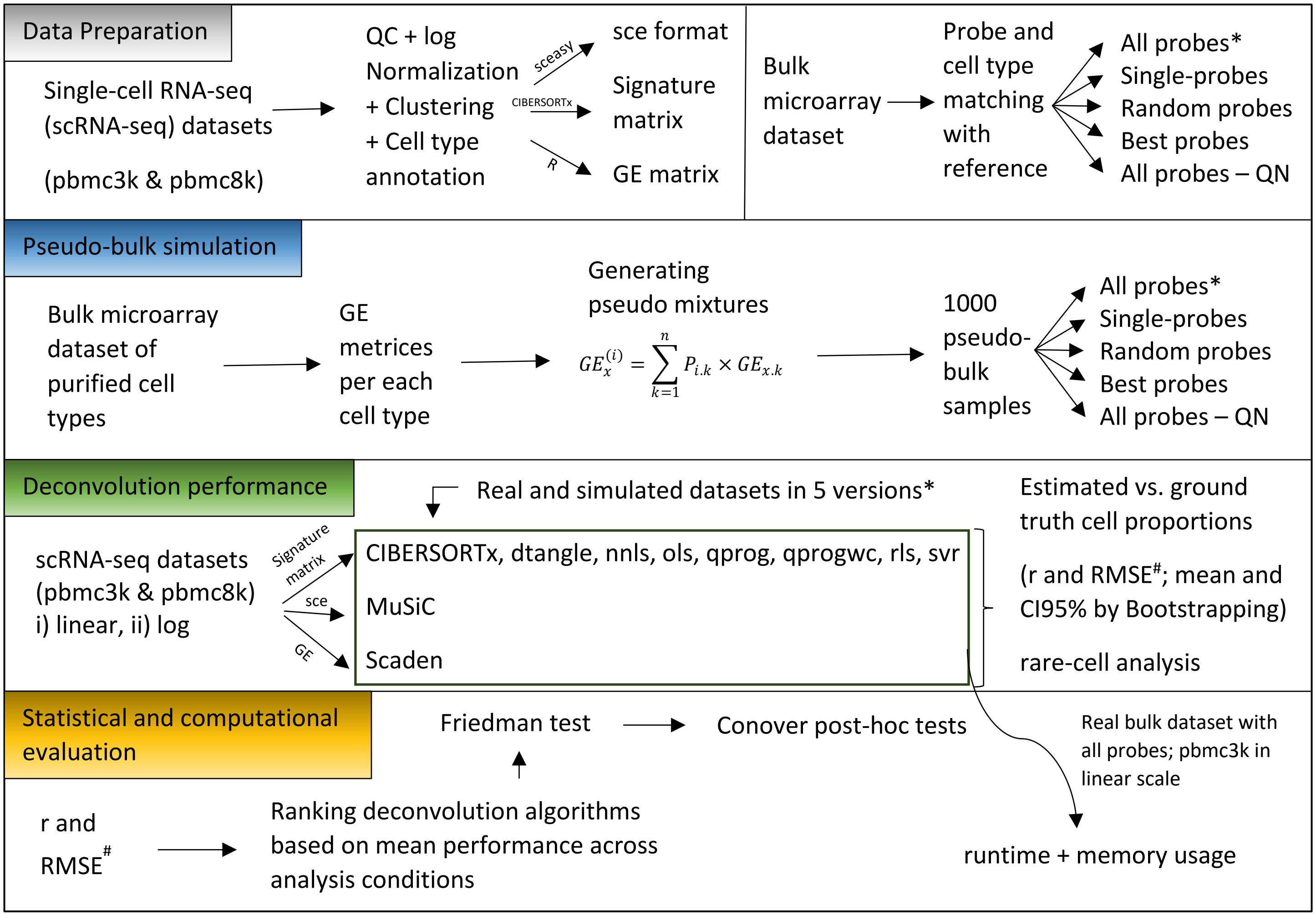

Figure S2. **The detailed analytical workflow of the study**. QC: quality control; scRNA-seq datasets will be converted to i) single-cell experiment (sce) format using sceasy R package, ii) signature matrix using CIBERSORTx, and iii) gene expression matrix (GE) using R base functions. QN: quantile-normalized; RMSE: root mean square error; CIBERSORTx: the extended version of cell type Identification by estimating relative subsets of transcription; dtangle: linear mixing model; nnls: non-negative least squares model; ols: ordinary least squares model; qprog and qprogwc: Quadratic programming without and with non-negativity and sum-to-one constraint; rls: re-weighted least squares; svr: support vector regression; MuSiC: multi-subject single cell deconvolution; Scaden: single cell-assisted deconvolutional deep neural networks.

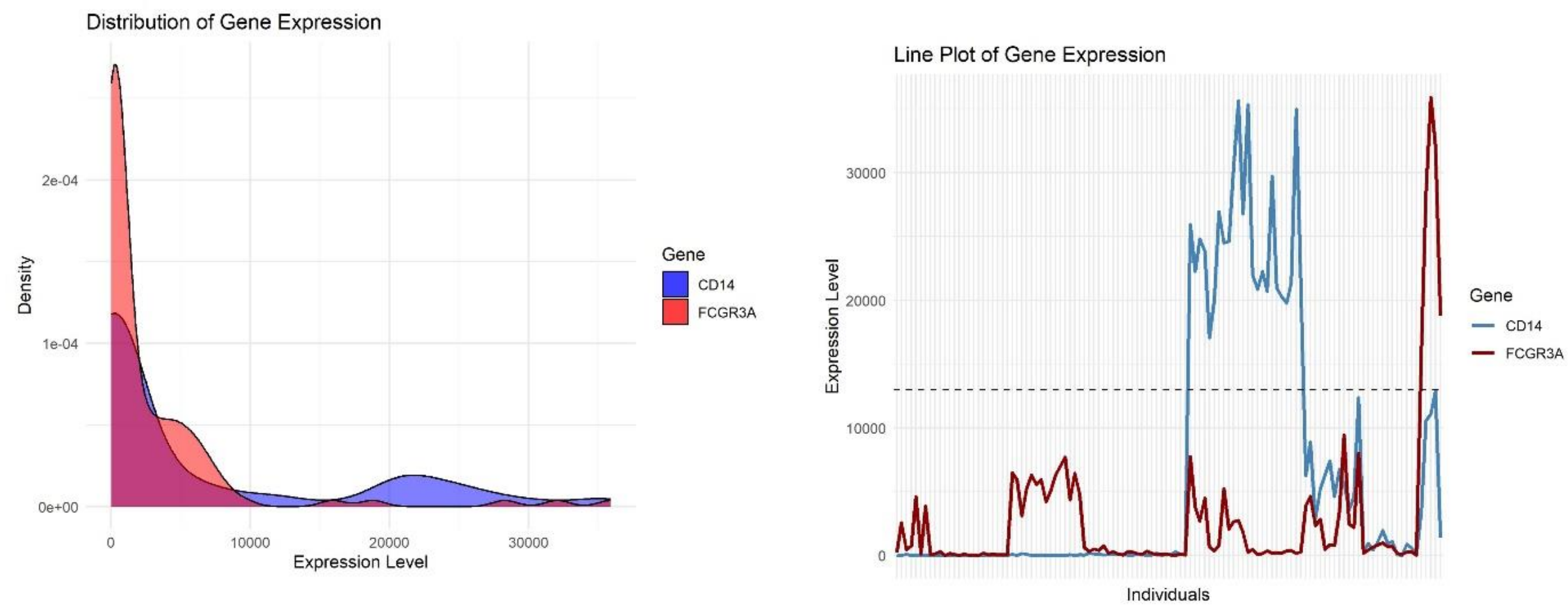

Figure S3 Separation of blood monocytes into two groups of CD14+ and FCGR3A+ monocytes. This was done based on *CD14* and *FCGR3A* gene expression levels in microarray data to match with the reference scRNAseq dataset. The left plot shows the distribution of these two genes' expression in microarray samples of monocyte cell type. The x-axis is the gene expression and y-axis is the density of cells. A threshold of 13000 was selected for separation of cells.

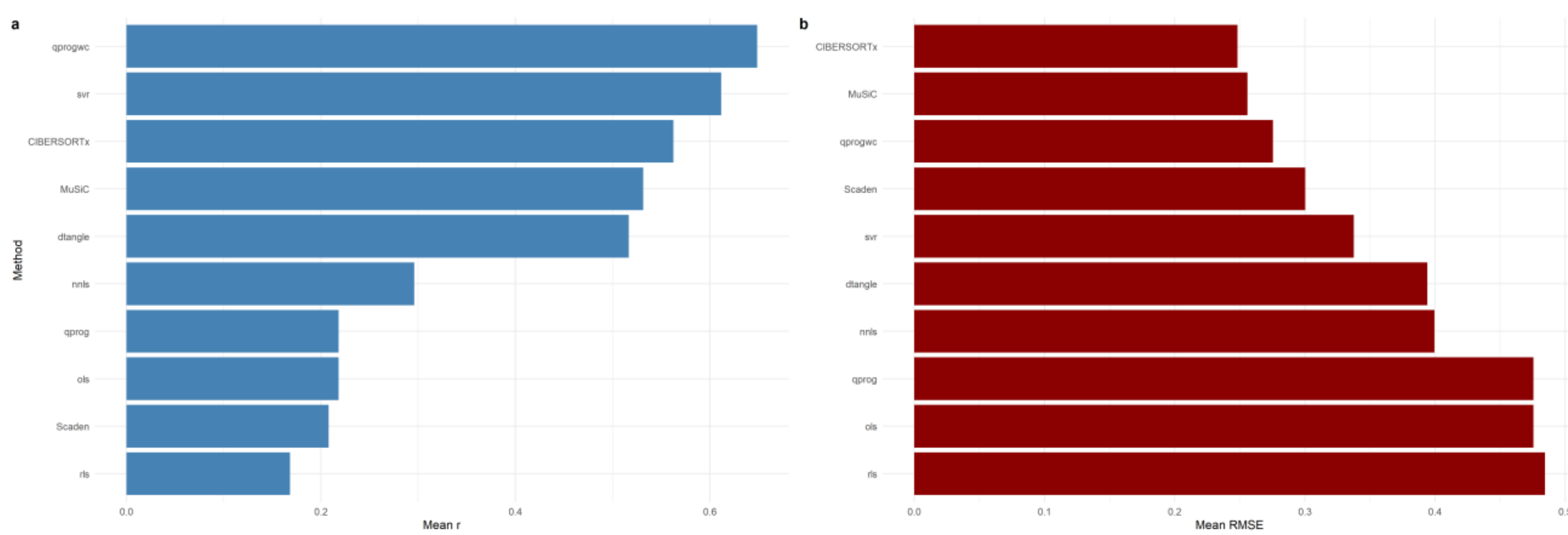

Figure S4. **Ranking of cell type deconvolution methods' performance**. This ranking is based on average performance metrics across different analyses conditions.

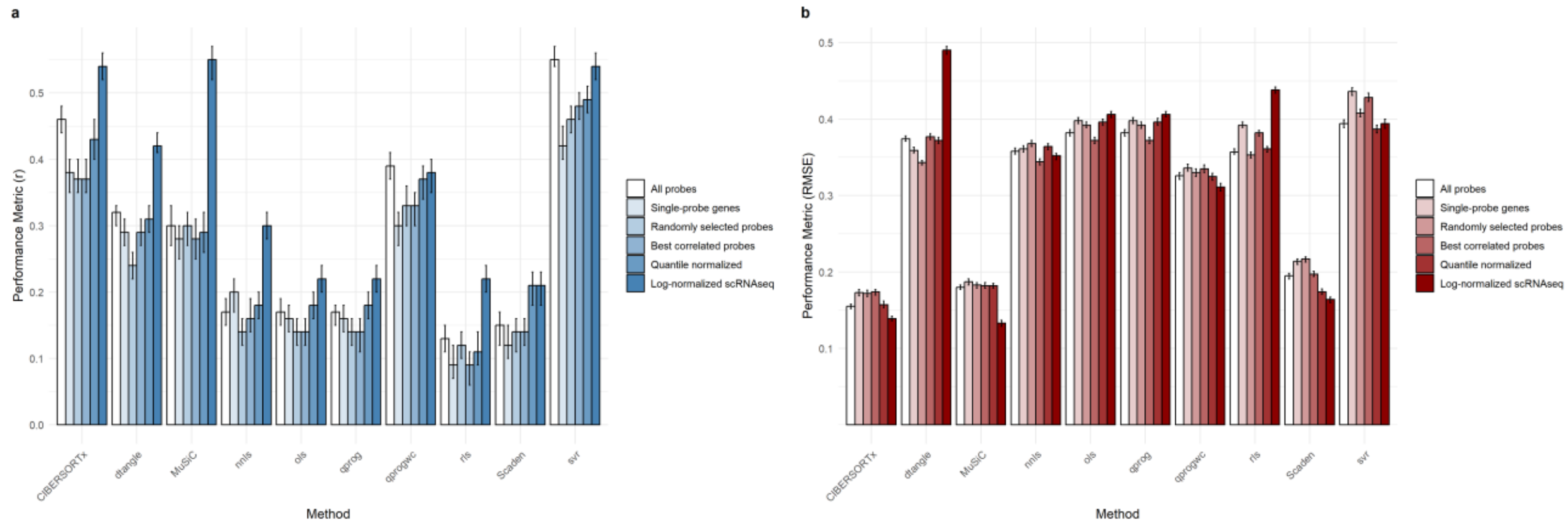

Figure S5. **Performance metrics of deconvolution algorithms when using 1000 simulated pseudo-bulk mixtures**. a) Correlation coefficients (Pearson r) and b) root mean squared error (RMSE) of real versus estimated cell type proportions. The error bars represent the 95% confidence interval (CI95%) calculated using the bootstrap method.

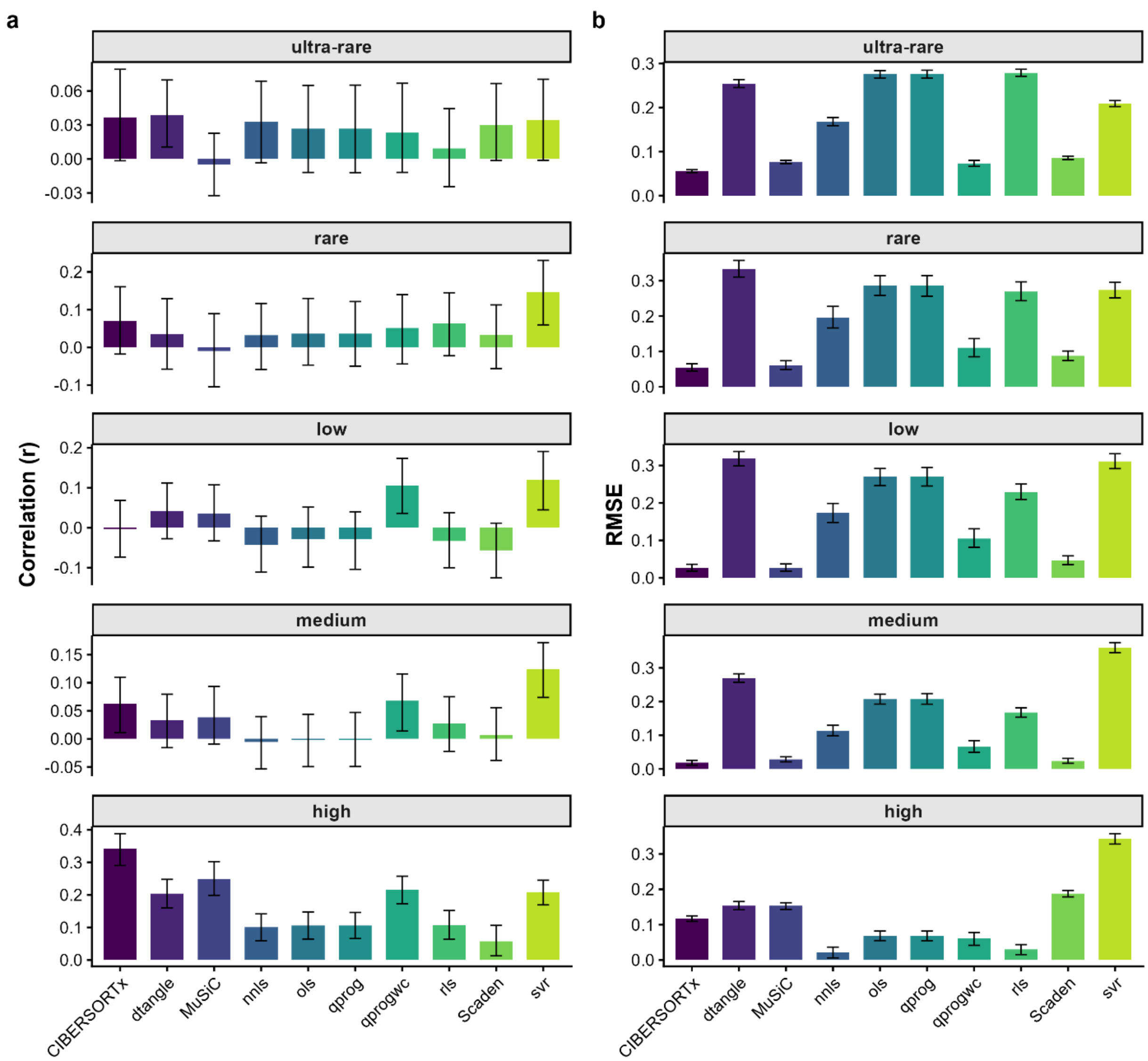

Figure S6. Performance comparison of deconvolution algorithms across different cell-type abundance groups in pseudo-bulk mixtures. a) Pearson correlation coefficient (*r*). b) Root mean squared error (RMSE). Cell-type abundance was categorized as Ultra-rare (<0.01), Rare (0.01–0.05), Low (0.05–0.10), Medium (0.10–0.20), and High (>0.20). The error bars represent the 95% confidence interval (CI95%) calculated using the bootstrap method.

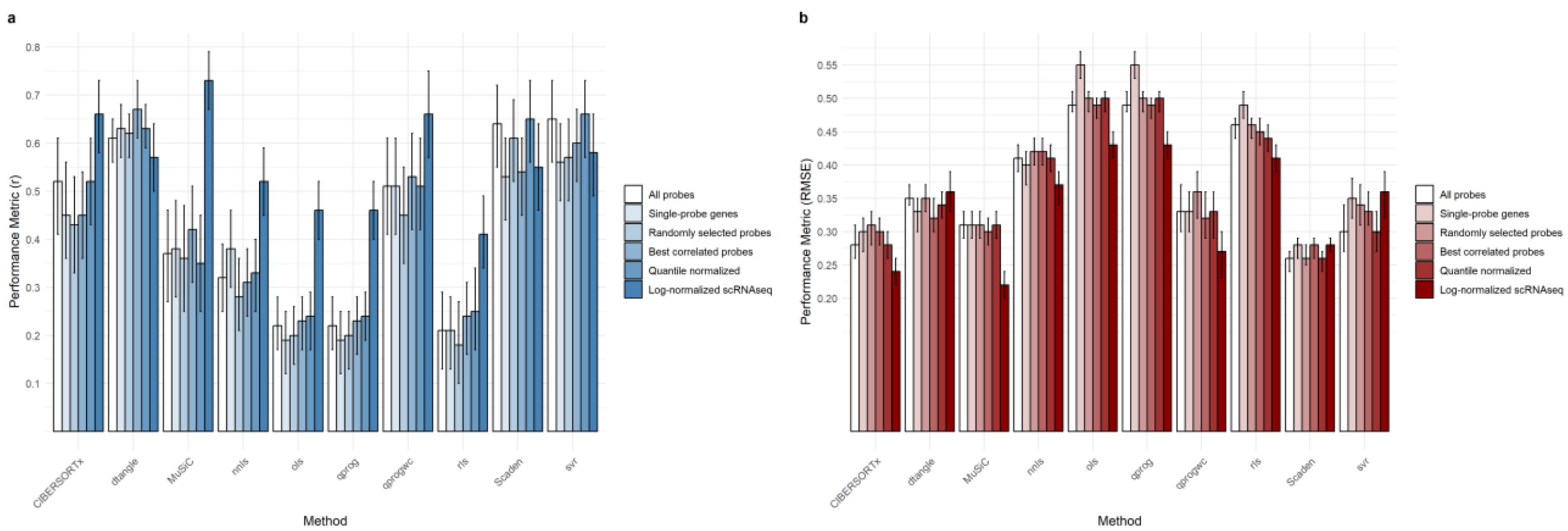

Figure S7. **Performance metrics of deconvolution algorithms when using an alternate single-cell reference panel.** This alternate reference was from a separate healthy donor and was sequenced with different technology. a) Correlation coefficients (Pearson r) and b) root mean squared error (RMSE) of real versus estimated cell type proportions. The error bars represent the 95% confidence interval (CI95%) calculated using the bootstrap method

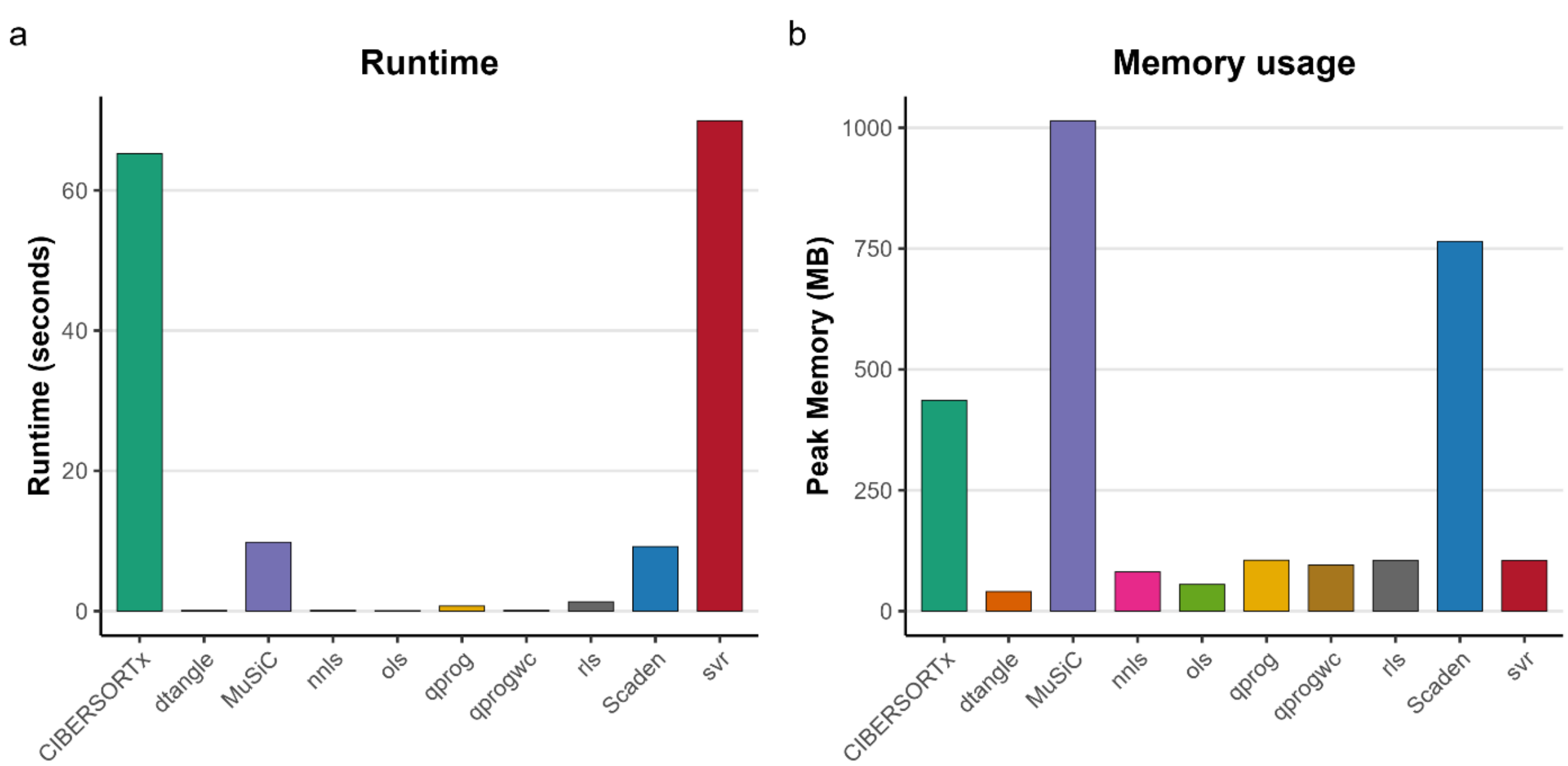

Figure S8. Computational efficiency of the prediction performance of deconvolution algorithms. Comparisons were made in terms of run time (a) and peak memory usage (b) under the same computational conditions.

Supplementary tables

Table S1. Friedman rank sum tests for performance metrics across deconvolution algorithms.

Metric	chi-squared	df	p-value
**r**	51.37	9	$5.96x10^{-8}$
**RMSE**	52.61	9	$3.47x10^{-8}$

Table S2. Conover post-hoc tests showing pairwise comparisons of performance (r) across deconvolution algorithms.

Method 1	Method 2	t value	Pr(>|t|)
dtangle	nnls	3.162	0.00331821
dtangle	ols	7.51	3.37E-09*
dtangle	qprog	7.51	3.37E-09*
dtangle	qprogwc	-9.092	2.12E-11*
dtangle	rls	11.859	7.20E-15*
dtangle	svr	-6.325	1.71E-07*
dtangle	CIBERSORTx	-3.953	0.00032843*
dtangle	MuSiC	-2.372	0.0241954
dtangle	Scaden	7.51	3.37E-09*
nnls	ols	4.348	9.98E-05*
nnls	qprog	4.348	9.98E-05*
nnls	qprogwc	-12.254	2.53E-15*
nnls	rls	8.696	7.33E-11*
nnls	svr	-9.487	6.25E-12*
nnls	CIBERSORTx	-7.115	1.24E-08*
nnls	MuSiC	-5.534	2.45E-06*
nnls	Scaden	4.348	9.98E-05*
ols	qprog	0	1
ols	qprogwc	-16.602	< 2.22e-16*
ols	rls	4.348	9.98E-05*
ols	svr	-13.835	< 2.22E-16*
ols	CIBERSORTx	-11.463	1.83E-14*
ols	MuSiC	-9.882	1.88E-12*
ols	Scaden	0	1
qprog	qprogwc	-16.602	< 2.22E-16*
qprog	rls	4.348	9.98E-05*
qprog	svr	-13.835	< 2.22E-16*
qprog	CIBERSORTx	-11.463	1.83E-14*
qprog	MuSiC	-9.882	1.88E-12*
qprog	Scaden	0	1
qprogwc	rls	20.95	< 2.22E-16*
qprogwc	svr	2.767	0.00943961
qprogwc	CIBERSORTx	5.139	8.98E-06*
qprogwc	MuSiC	6.72	4.60E-08*
qprogwc	Scaden	16.602	< 2.22E-16*
rls	svr	-18.183	< 2.22E-16*
rls	CIBERSORTx	-15.811	< 2.22E-16*

Method 1	Method 2	t value	Pr(>|t|)
rls	MuSiC	-14.23	< 2.22E-16*
rls	Scaden	-4.348	9.98E-05*
svr	CIBERSORTx	2.372	0.0241954
svr	MuSiC	3.953	0.00032843*
svr	Scaden	13.835	< 2.22E-16*
CIBERSORTx	MuSiC	1.581	0.12948257
CIBERSORTx	Scaden	11.463	1.83E-14*
MuSiC	Scaden	9.882	1.88E-12*

* Conover significant p-values after correction for multiple testing (P < 0.001 i.e., 0.05/45 with 45 as the number of pairwise tests)

Table S3. Conover post-hoc tests showing pairwise comparisons of performance (RMSE) across deconvolution algorithms.

Method 1	Method 2	t value	Pr(>|t|)
dtangle	nnls	0	1
dtangle	ols	-7.618	1.76E-09*
dtangle	qprog	-7.618	1.76E-09*
dtangle	qprogwc	11.426	1.39E-14*
dtangle	rls	-9.25	9.77E-12*
dtangle	svr	4.897	1.58E-05*
dtangle	CIBERSORTx	17.411	< 2.22E-16*
dtangle	MuSiC	15.235	< 2.22E-16*
dtangle	Scaden	8.162	3.23E-10*
nnls	ols	-7.618	1.76E-09*
nnls	qprog	-7.618	1.76E-09*
nnls	qprogwc	11.426	1.39E-14*
nnls	rls	-9.25	9.77E-12*
nnls	svr	4.897	1.58E-05*
nnls	CIBERSORTx	17.411	< 2.22E-16*
nnls	MuSiC	15.235	< 2.22E-16*
nnls	Scaden	8.162	3.23E-10*
ols	qprog	0	1
ols	qprogwc	19.044	< 2.22E-16*
ols	rls	-1.632	0.11468818
ols	svr	12.514	6.65E-16*
ols	CIBERSORTx	25.029	< 2.22E-16*
ols	MuSiC	22.853	< 2.22E-16*
ols	Scaden	15.779	< 2.22E-16*
qprog	qprogwc	19.044	< 2.22E-16*
qprog	rls	-1.632	0.11468818
qprog	svr	12.514	6.65E-16*
qprog	CIBERSORTx	25.029	< 2.22E-16*
qprog	MuSiC	22.853	< 2.22E-16*
qprog	Scaden	15.779	< 2.22E-16*
qprogwc	rls	-20.676	< 2.22E-16*
qprogwc	svr	-6.529	6.75E-08*

Method 1	Method 2	t value	Pr(>|t|)
qprogwc	CIBERSORTx	5.985	4.22E-07*
qprogwc	MuSiC	3.809	0.00049807*
qprogwc	Scaden	-3.265	0.00236063
rls	svr	14.147	< 2.22E-16*
rls	CIBERSORTx	26.661	< 2.22E-16*
rls	MuSiC	24.485	< 2.22E-16*
rls	Scaden	17.411	< 2.22E-16*
svr	CIBERSORTx	12.514	6.65E-16*
svr	MuSiC	10.338	3.55E-13*
svr	Scaden	3.265	0.00236063
CIBERSORTx	MuSiC	-2.176	0.03820741
CIBERSORTx	Scaden	-9.25	9.77E-12*
MuSiC	Scaden	-7.073	1.08E-08*

* Conover significant p-values after correction for multiple testing (P < 0.001 i.e., 0.05/45 with 45 as the number of pairwise tests)

Supplementary methods for pre-processing input data

Method S1. Pre-processing details of scRNAseq reference panel for cell type deconvolution algorithms

The Scaden algorithm required the h5ad format of the single cell reference file which was generated by the 'simulate' command from its own software. The MuSiC method needed a SingleCellExperiment R object which was obtained from the Seurat object using convertFormat() function from the sceasy R package[S1]. For other cell type deconvolution techniques a signature matrix was needed, which was built using the CIBERSORTx[S2] algorithm.

Method S2. Selection strategy of best correlating probes for microarray data

Based on an adapted strategy of Võsa et al[S3], we first selected the intersect of genes in the bulk microarray dataset and the scRNAseq reference panel. Then we designated those with only a single probe in microarray as the 'base gene set'. Second, we calculated the correlations of each probe of multi-probe genes with the base gene set in bulk as well as reference datasets, separately. Third, for each multi-probe gene we selected the representative probe as the probe with highest similarity (read correlation) of correlations with the base gene set in bulk dataset and those in reference dataset.

References

S1. Cakir, B., Prete, M., Huang, N., van Dongen, S., Pir, P., and Kiselev, V.Y. (2020). Comparison of visualization tools for single-cell RNAseq data. NAR Genomics Bioinforma. *2*, lqaa052. https://doi.org/10.1093/nargab/lqaa052.

S2. Newman, A.M., Steen, C.B., Liu, C.L., Gentles, A.J., Chaudhuri, A.A., Scherer, F., Khodadoust, M.S., Esfahani, M.S., Luca, B.A., Steiner, D., et al. (2019). Determining cell type abundance and expression from bulk tissues with digital cytometry. Nat. Biotechnol. *37*, 773–782. https://doi.org/10.1038/s41587-019-0114-2.

S3. Võsa, U., Claringbould, A., Westra, H.-J., Bonder, M.J., Deelen, P., Zeng, B., Kirsten, H., Saha, A., Kreuzhuber, R., Yazar, S., et al. (2021). Large-scale cis- and trans-eQTL analyses identify thousands of genetic loci and polygenic scores that regulate blood gene expression. Nat. Genet. *53*, 1300–1310. https://doi.org/10.1038/s41588-021-00913-z.
